# Supplementary material for: Magnon spectroscopy in the electron microscope
Source: Nature. 2025 Jul 23;644(8075):83–8. doi: 10.1038/s41586-025-09318-y (PMC12328233; doi:10.1038/s41586-025-09318-y)
Supplement: Supplementary file 1 — This file contains Supplementary Notes 1–7, Supplementary Figs. 1–8 and Supplementary References. [file 41586_2025_9318_MOESM1_ESM.pdf]

nature portfolio

https://doi.org/10.1038/s41586-025-09318-y

Supplementary information

Magnon spectroscopy in the electron microscope

In the format provided by the authors and unedited

Supplementary Information

Magnon spectroscopy in the electron microscope

Demie Kepaptsoglou[1,2,3*†], José Ángel Castellanos-Reyes[4†], Adam Kerrigan[2,3], Júlio Alves do Nascimento[2,3], Paul M. Zeiger[4], Khalil El hajraoui[1,2], Juan Carlos Idrobo[5,6], Budhika G. Mendis[7], Anders Bergman[4], Vlado K. Lazarov[2,3], Ján Rusz[4*], and Quentin M. Ramasse[1,8,9*]

[1]*SuperSTEM Laboratory, SciTech Daresbury Campus, Daresbury, WA4 4AD, UK.*
[2]*School of Physics, Engineering and Technology, University of York, Heslington, YO10 5DD, UK.*
[3]*JEOL NanoCentre, University of York, Heslington, YO10 5DD, UK.*
[4]*Department of Physics and Astronomy, Uppsala University, Box 516, Uppsala, 75120, Sweden.*
[5]*Materials Science and Engineering Department, University of Washington, Seattle, WA 98195, USA.*
[6]*Physical & Computational Sciences Directorate, Pacific Northwest National Laboratory, Richland, WA 99354, USA.*
[7]*Department of Physics, Durham University, Durham, DH1 3LE, UK.*
[8]*School of Chemical and Process Engineering, University of Leeds, Leeds, LS2 9JT, UK.*
[9]*School of Physics and Astronomy, University of Leeds, Leeds, LS2 9JT, UK.*

*Corresponding author(s). E-mail(s): dmkepap@superstem.org; jan.rusz@physics.uu.se; qmramasse@superstem.org.
[†]These authors contributed equally to this work.

Contents

Supplementary Note 1: Intensity scaling 2
Supplementary Fig. 1 | Momentum-resolved vibrational EELS measurements of NiO. 3
Supplementary Note 2: Background subtraction 4
Supplementary Fig. 2 | Robustness of magnon signal against background-subtraction models............ 5
Supplementary Note 3: Data accumulation and signal-to-noise 6
Supplementary Table 1 | Calculated SNR for partial and complete datasets. 7
Supplementary Fig. 3 | Frame accumulation in ω-**q** maps along the 220 and 002 rows of reflections.... 8
Supplementary Note 4: Experimental broadening............ 9
Supplementary Fig. 4 | Broadened simulated magnon EELS dispersions............ 10
Supplementary Note 5: Additional datasets 11
Supplementary Fig. 5 | ω-**q** map along Γ → M **q**-path. 11
Supplementary Note 6: Spatially resolved spectral variations 12
Supplementary Fig. 6 | Spatially resolved magnon EELS measurements across a NiO thin film. 13
Supplementary Note 7: Inelastic magnon scattering calculations for a NiO slab model 14
Supplementary Fig. 7 | Spatial dependence of magnon scattering across a NiO slab. 15
Supplementary Fig. 8 | Comparison between experiment and theory across a NiO thin film.............. 15
Supplementary references............ 16

Supplementary Note 1: Intensity scaling

Due to the 1/*E* dependence of the classical model for the harmonic oscillator strength (where *E* is the energy of the oscillation) [70], phonon scattering data is often displayed with the observed experimental intensity scaled by the energy loss *E.* This offers a more direct comparison to *ab initio* calculations (see, e.g., neutron inelastic scattering data in ref. [71]). Accounting for the energy-dependent occupancy of phonon excitations with a Bose-Einstein model (assuming zero chemical potential), an additional factor of 1/*E* appears in the oscillator strength model when approximating $1/[\exp(E/k_BT)-1] \sim k_BT/E$, if $E << k_BT$, where k_B is the Boltzmann constant, and *T* is the temperature. As a result, a total scaling of the experimental scattered intensity by E^2 can be justified at low energy losses.

A number of research groups have therefore proposed displaying high-energy-resolution EELS data after scaling by the square of the energy loss, that is, displaying [intensity × (energy-loss)^2] *vs.* (energy-loss) [72]. This scaling by E^2 should strictly only be valid at energies well below k_BT, *i.e.* a few meV at room temperature. Nevertheless, even when applied across larger energy-loss windows, this strategy provides a useful means to enhance the visibility of weaker intensity features above the decaying zero-loss-peak (ZLP) tail in the meV range, while avoiding possible errors and subjectivity in background fitting. Considered as a pure ‘data scaling’ strategy (as would be a logarithmic intensity display), it helps to visualise signals with vastly different intensity levels on the same panel, including in close vicinity to the ZLP. Supplementary Fig. 1 illustrates the effectiveness of this data scaling approach in revealing more clearly the presence of the magnon scattering branches above the phonon energy range in the full ω-**q** maps for NiO.

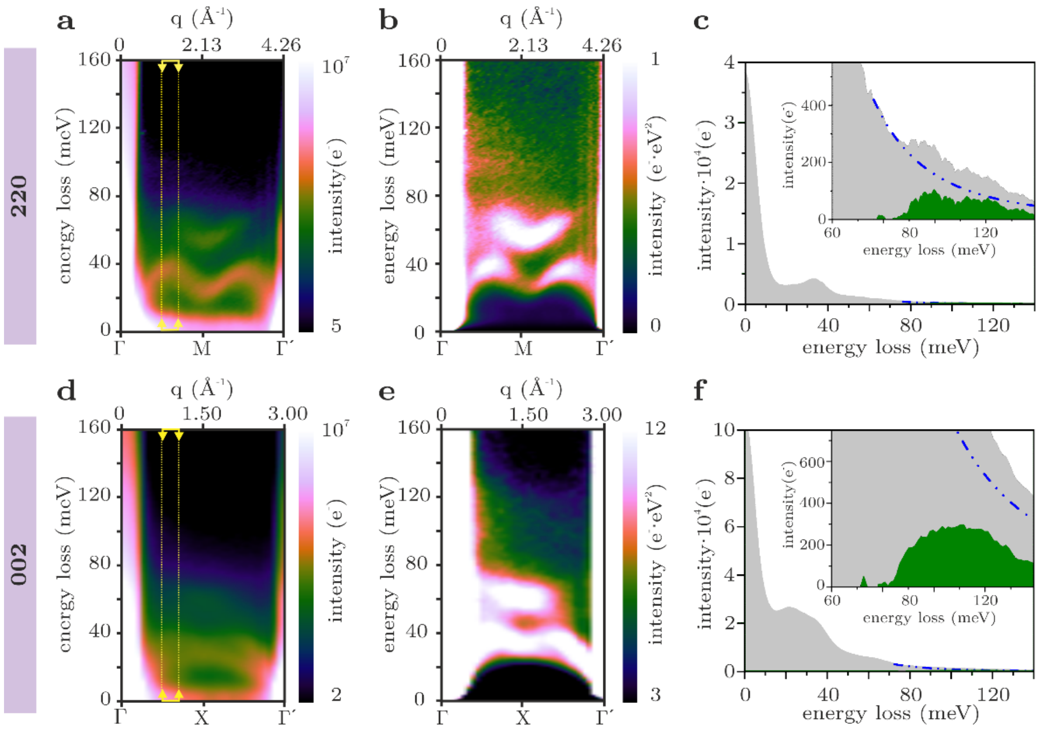

Supplementary Fig. 1 | Momentum-resolved vibrational EELS measurements of NiO. *a,d.* *As-acquired ω-**q** maps along the 220 and 002 rows of reflections, respectively, showing the dispersion of the NiO LA and LO phonon branches, displayed on a logarithmic intensity scale.* ***b,e.*** *ω-**q** maps along the same reciprocal space directions scaled by the square of the energy loss, E^2.* ***c,f.*** *Integrated spectra at the specific momentum positions marked by arrows in panels **a,d**. Inset: spectral intensity (green shaded areas) after background subtraction (the blue dotted lines illustrate the background model, while the shaded grey area is the unprocessed intensity).*

Supplementary Note 2: Background subtraction

The use of a power-law function to remove any decaying background is one of the most common approaches in EELS, as it models population statistics quite effectively and appears to be relatively robust, even in the vibrational EELS range [73, 74]. However, the stability of the model across neighbouring pixels in 2-dimensional datasets like ω-$\mathbf{q}$ maps can be poor when the energy fitting window is narrow. This is often the case for very low energy losses close to the zero-loss peak tail, or for signals superimposed on the tail of other losses close in energy. In turn, this can lead to subjective results depending on a given user's fitting window selection. As a result, background subtraction is often employed on a case-by-case basis, considering various background options, which can sacrifice physical justification to improve visibility [75].

Here, we explore different fitting options for the removal of the LO phonon decaying background immediately before the energy range where our simulations predict the presence of the magnon signal. Supplementary Figure 2 presents the ω-$\mathbf{q}$ maps along the 220 systematic rows of reflections, using first-order logarithmic-polynomial and power-law models for comparable energy windows. In both cases, the resulting signal shows a remarkable qualitative resemblance to the calculated curves in Figure 3 of the main manuscript, demonstrating the robustness of the signal against the choice of background model. However, the use of the power-law background appears to yield noisier maps, as it is more susceptible to variations in phonon intensity tail across the narrow fitting energy window, resulting in some negative values, particularly on the weaker M→Γ' magnon branch.

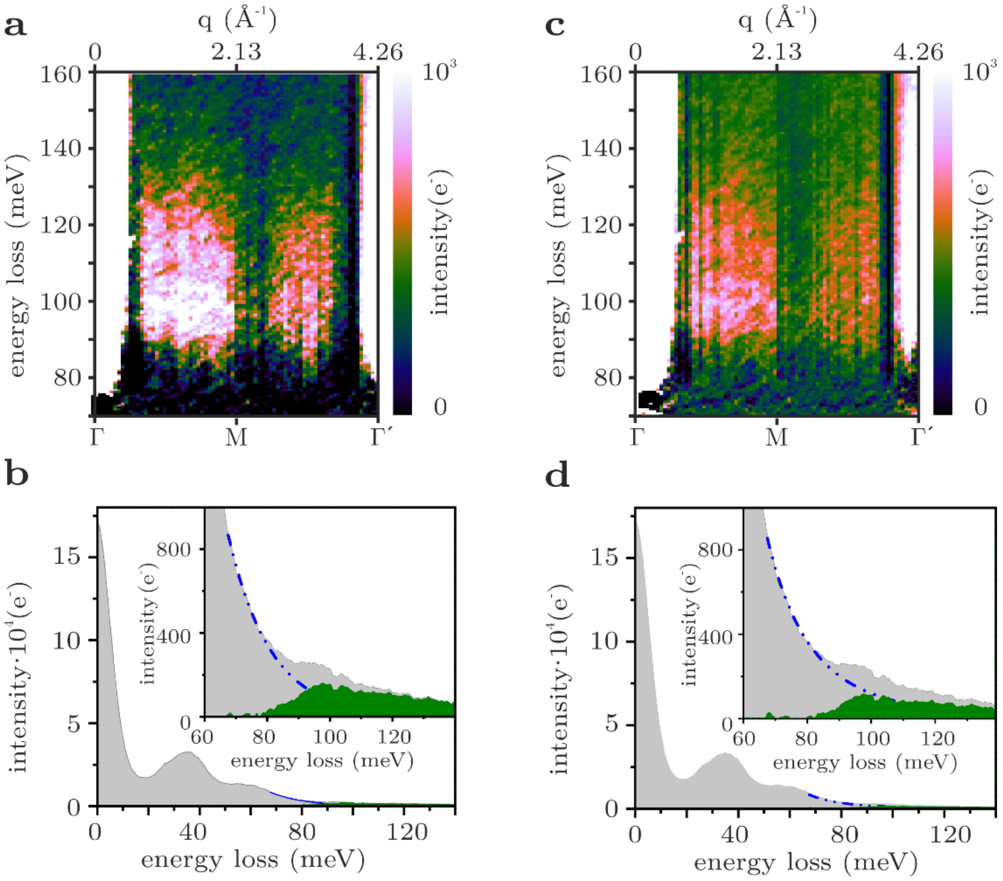

Supplementary Fig. 2 | Robustness of magnon signal against background-subtraction models. *Background-subtracted ω-**q** maps along Γ→M→Γ' and corresponding extracted spectra (inset: signal after background subtraction, green shaded area; the dotted blue lines are the decaying background model, and the shaded grey area is the unprocessed spectral intensity) using: **(a,b)** a first-order log-polynomial background model and **(c,d)** a power-law model.*

Supplementary Note 3: Data accumulation and signal-to-noise

As discussed in the Methods section of the paper, the ω-**q** EELS datasets were acquired as multiple frames covering the entire two-dimensional extent of the spectrometer's camera. A typical single dataset comprised 15,000 frames (limited by data file size), which were subsequently aligned using rigid image registration to account for possible instabilities, and integrated. The final data presented are sums of multiple such integrated datasets acquired consecutively at the exact same sample position, on the same day and under identical experimental conditions, with no other post-processing applied. The signal accumulation was used to improve the signal-to-noise ratio and the visibility of the inherently weak magnon signal in the final dataset.

Datasets were not averaged over several days' acquisition, over series acquired at slightly different locations, or across datasets involving other experimental changes, to maintain the nanometre scale resolution of the experiments on the very same sample area.

Supplementary Figure 3 shows ω-**q** maps along the 220 and 002 rows of systematic reflections; panels **a** and **c** correspond to sums of 90,000 (6 x 15,000, SNR = 22) and 60,000 (4 x 15,000, SNR = 59) individual camera frames, respectively, while panels **b** and **d** consist of partial frame-integrated datasets from **a** and **c,** comprising only the first 30,000 (SNR = 10.7) and 15,000 (SNR = 16) frames from the respective series. The smaller number of frames required to obtain comparable SNR in the case of the 002 data is due to a tip change occurring between the two sets of experiments, resulting in higher probe currents available at otherwise similar electron optical settings. The magnon dispersion bands and their separation in the momentum direction are clearly discernible in both partial datasets, albeit with higher noise levels as quantified by the calculated SNRs, which remain well above 10 even with less data accumulation.

This illustrates the effect of large signal series accumulation, with a need to balance higher overall SNR with the potential for partial smearing of the final signal fine structure, due to unintended experimental instabilities through the hours-long acquisitions, that can be challenging to mitigate in post-processing. This is particularly evident along the Γ → X **q**-path (002), where the magnon intensity lobes in the dispersion band are clearly separated in panel **d**, with the intensity dropping to zero near X (as predicted by simulations). In contrast, the lobe separation and momentum variation are less evident in the larger dataset averaged over more frames, shown in panel **c**; however, this dataset has higher signal-to-noise and signal-to-background, providing an unambiguous confirmation of the presence of spectral intensity in the relevant energy window.

Here, the signal-to-noise ratio (SNR) of a given magnon dispersion diagram is defined as $SNR = \frac{\mu(d\omega,dE)}{\sigma(d\omega,dE)}$ where $\mu(d\omega, dE)$ is the mean spectral intensity in the magnon energy window after

background subtraction, in otherwise unprocessed spectral data. The noise $\sigma(d\omega, dE\ )$ is in turn is estimated as the standard deviation of the recorded signal in an energy window of the same size, beyond any predicted magnon or phonon contribution (above 200 meV).

For signals whose distribution follows Poisson statistics, a more exact mathematical definition of the SNR from counting statistics can be used. While the EELS detector used here is not strictly a pure 'electron counter', its noise characteristics are known to be nearly Poisson-limited [31] and thus the following calculations were also taken into consideration for completeness (and the pixel-wise definition of the standard deviation below as the main source of counting error was used to derive the confidence bands displayed in Figure 2 or the main manuscript).

- $\mathrm{SNR} = \frac{\mathrm{M(d\omega,dE)}}{\sqrt{\mathrm{N(d\omega,dE)}}}$, where $\mathrm{N(d\omega, dE)}$ and $\mathrm{M\ (d\omega, dE)}$ are the mean spectral intensities in the magnon energy window before and after background subtraction, respectively, in otherwise unprocessed spectral data.
- $\mathrm{SNR} = \frac{\mathrm{IM(d\omega,dE)}}{\sqrt{\mathrm{IN(d\omega,dE)}}}$, where $\mathrm{IN(d\omega, dE)}$ and $\mathrm{IM\ (d\omega, dE)}$ are the integrals of the spectral intensities in the magnon energy window before and after background subtraction, respectively, in otherwise unprocessed spectral data.

All these are summarised in Supplementary Table 1 and clearly demonstrate how frame accumulation results in higher SNR irrespective of the metric used, while all values suggest that the signal is clearly above noise level.

Supplementary Table 1 | Calculated SNR for partial and complete datasets. *The calculated values correspond to panels presented in Fig. 2 and Supplementary Figure 3.*

	**220**		**002**	
**SNR**	30k frames	90k frames	15k frames	60k frames
$\boldsymbol{\frac{\mu(d\omega, dE)}{\sigma(d\omega, dE)}}$	10.7	22	16	59
$\boldsymbol{\frac{M(d\omega, dE)}{\sqrt{N(d\omega, dE)}}}$	2	3	2	10
$\boldsymbol{\frac{IM(d\omega, dE)}{\sqrt{IN(d\omega, dE)}}}$	81	134	44	222

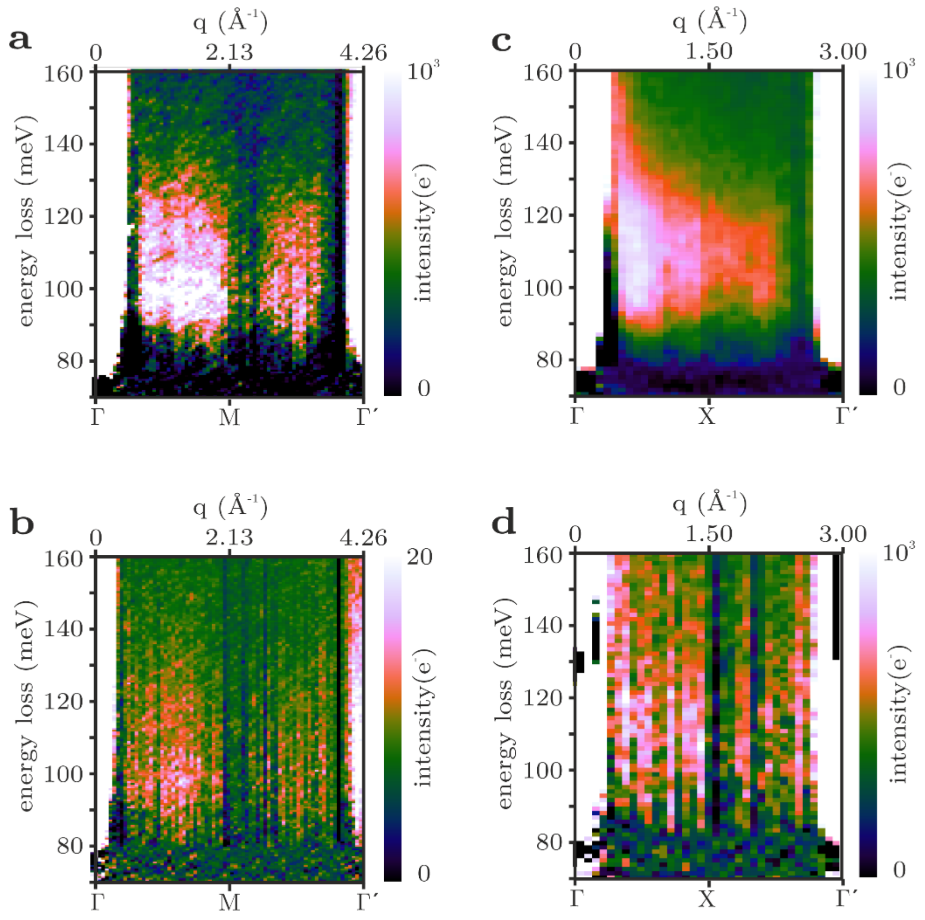

Supplementary Fig. 3 | Frame accumulation in ω-q maps along the 220 and 002 rows of reflections. ***a*****,*****c*****.** *ω-**q** magnon maps corresponding to sums of 90,000 (SNR = 22) and 60,000 (SNR = 59) individual camera frames, respectively, corresponding to 112.5 minutes and 75 minutes of live-beam acquisition respectively (the effective experimental time is longer to account for data transfer of partial datasets between bursts of 15,000 frames).* ***b*****,*****d*****.** *Partial datasets from **a** and **c** comprising 30,000 (SNR = 10.7) and 15,000 (SNR = 16) frames only (corresponding to live-beam times of 37.5 minutes and 18.75 minutes), respectively.*

Supplementary Note 4: Experimental broadening

The impact of finite experimental resolution in momentum and energy on the separation of the magnon dispersion bands was illustrated by numerically broadening the simulated magnon EELS dispersion diagrams along the Γ → M and Γ → X **q**-paths to match experimental limitations. This was achieved by convolution with a top-hat function of 0.7 $Å^{-1}$ width along the momentum axis (mimicking the momentum-selecting slit), and with a Gaussian of 11 meV full-width at half-maximum along the energy-dispersive axis (reflecting the effective energy resolution of the experiment in the 002 case, with the probe going through the sample after integration of 90,000 frames).

As expected, due to the shorter reciprocal space distance, the broadening has more apparent impact on the data acquired along the Γ → X **q**-path; the magnon peaks appear to merge into a more continuous lobe of intensity, reflecting the experimental data where, as discussed in the main manuscript, the two peaks are hard to separate on either side of the X-point.

The match in the Γ → M direction, where the Brillouin zone vertices are spaced further apart so the magnon lobes are still resolved, is also more 'pleasing' visually (albeit less defined) after this forward convolution.

We note that the theoretical framework for magnon-EELS simulations is still being actively developed. At present, these approaches do not yet incorporate magnon-phonon coupling or fully capture the complex influence of phononic backgrounds. A fully coupled magnon-phonon framework, which is not yet available, would be required to reproduce the complete spectral landscape, including the intricate interplay between signal and background, as well as to address effects such as background subtraction, noise, and broadening in a more systematic and quantitative way. Nonetheless, the uncoupled simulations presented here, especially after forward convolution to mimic experimental broadening, enable a clear identification of magnonic features based on their dispersion behaviour and alignment with Brillouin zone boundaries and high-symmetry points—providing compelling evidence for the interpretation of the experimentally observed excitations. These theoretical developments are ongoing and will be the subject of future communications and publications.

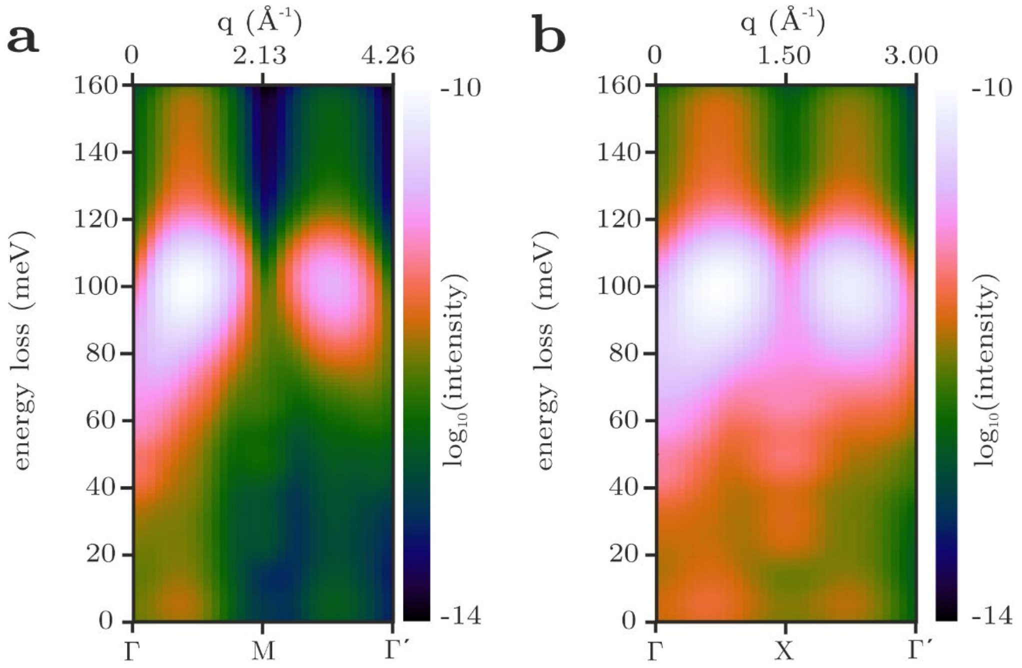

Supplementary Fig. 4 | Broadened simulated magnon EELS dispersions. *Simulated ω-**q** magnon EELS dispersions (intensity displayed on a logarithmic colour scale) for the Γ → M **(a)** and Γ → X **(b)** directions, respectively, after convolution with a top-hat function of 0.7 $Å^{-1}$ width along the momentum axis and a Gaussian of 11 meV-full-width at half-maximum along the energy-dispersive axis.*

Supplementary Note 5: Additional datasets

Supplementary Figure 5 shows an additional, independent dataset acquired along the Γ → M **q**-path corresponding to 75,000 individual (5 x 15,000) camera frames, where both the (LA and TA) phonon (Supplementary Fig. 5a) and magnon (Supplementary Fig. 5b) bands are symmetrically resolved on either side of the Γ-point.

This demonstrates the reproducibility of the results, as the dataset was acquired on a different day, and in a different area of the sample from that shown in the main manuscript, Fig. 2. The wider momentum window presented in Supplementary Fig. 5 highlights symmetric data on either side of Γ, but due to the wider angular (momentum) range, some asymmetric energy resolution loss due to spectrometer aberrations along the length of the momentum-selecting slit results in minor feature blurring in the Γ-M-Γ' direction, compared to Γ-M-Γ''. On the right side of the Γ-point, the phonon bands are not as well-resolved, and the magnon lobes not as clearly separated. However, the phonon and magnon bands are clearly recognisable on both sides.

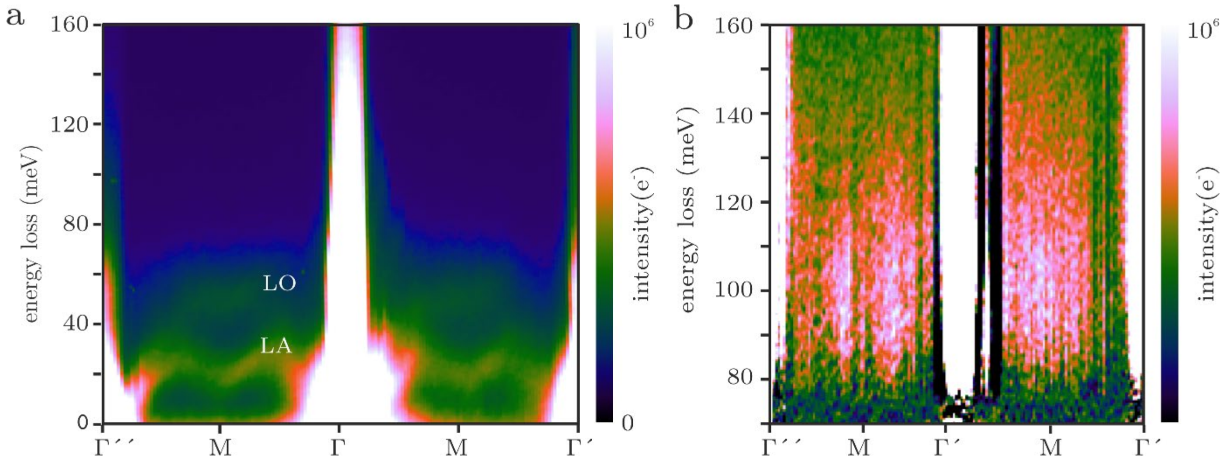

Supplementary Fig. 5 | ω-q map along Γ → M q-path. ***a.*** *ω-**q** map corresponding to 75,000 (5 x 15,000) individual camera frames.* ***b.*** *Background-subtracted magnon dispersion diagram showing a symmetric dispersion of the magnon signal across the Γ-point.*

Supplementary Note 6: Spatially resolved spectral variations

Spectral analysis of the spatially resolved dark-field EELS experiments shows a well-defined magnon peak at 100 meV (Supplementary Fig. 6c) throughout the NiO thin film. The intensity of this peak is significantly suppressed when the probe is positioned inside the NiO film but within a sub-nm distance of the interface with the (non-magnetic) YSZ substrate on one side, and close to the hole created immediately above the NiO thin film during the FIB sample preparation procedure on the other. The peak shape is also altered, with a slight, but noticeable broadening of the main peak near the thin film edge and the interface. Probe propagation could play a role in these observed signal changes, as mentioned in the main text in the discussion of Fig. 4. However, magnon EELS simulations for a thin slab of NiO also predict a similar effect in the vicinity of the slab's surfaces (Supplementary Fig. 7 and Supplementary Fig. 8). Although at 20 nm wide the NiO thin film is still "thick", this broadening may be linked to the appearance of confinement-related additional softer magnon modes in the ultra-thin film limit [76], a phenomenon we plan to study in future work.

Supplementary Figure 6d shows the background-subtracted NiO magnon spectrum plotted alongside spectra from the YSZ substrate and a reference spectrum acquired in identical conditions from the carbon protective layer deposited prior to FIB sample preparation. The preparation procedure had resulted in the formation of a hole (vacuum) immediately above the NiO film in the spectrum image region-of-interest, but some remaining carbon from the protective cap remained present far above the surface in other, extended-range data. This allows for a direct and self-consistent comparison.

The 'carbon' spectrum displays a characteristic, strong vibrational signature above 150 meV (attributed to C-C vibrational and C-O modes). This can also be seen in the NiO 'surface' spectrum (Supplementary Fig. 6c), as some small amount of carbon may have remained at the very surface of the film. Similarly, some faint intensity at >150 meV, *i.e.* in the range corresponding to carbon, and which could have arisen from minor contamination build-up during the hours-long acquisition, can be seen in some of the spectra in Supplementary Fig. 6c ('bulk') and 6d ('NiO' and 'YSZ'). These are distinct in energy range and spectral shape, and of such low intensity compared to any other contribution, that carbon can be excluded with confidence as a spurious contribution to the magnon signal.

YSZ does not sustain magnons, but exhibits a main optical phonon band around 76 meV corresponding to O vibrational modes [77], which we observe in our spectra and is distinct in shape and energy from the NiO magnon peak. In future work, spatially resolved dispersion diagrams across interfaces (using a small convergence angle and slit EELS aperture) [78] will provide a way to study interface-induced modifications to magnon dispersions. Here, given the non-magnetic nature of YSZ, such an experiment would simply show the individual contributions of YSZ (phonons only) or NiO (phonons and magnon).

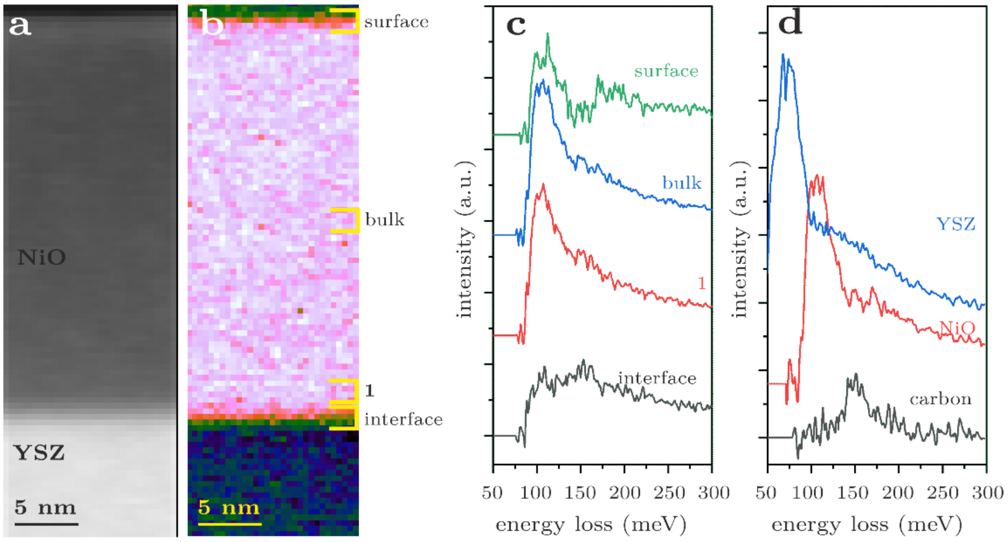

Supplementary Fig. 6 | Spatially resolved magnon EELS measurements across a NiO thin film. ***a.*** *Asymmetric (displaced) annular-dark-field Image (a-ADF) acquired during EELS measurements.* ***b.*** *Integrated intensity map of the magnon peak.* ***c.*** *Background subtracted magnon spectra from areas of the NiO film marked in* ***(b)****.* ***d.*** *Background-subtracted NiO magnon spectrum plotted alongside spectra from the YSZ substrate and a reference spectrum from the C protective layer of the FIB-prepared sample, respectively, as indicated.*

Supplementary Note 7: Inelastic magnon scattering calculations for a NiO slab model

To investigate the effects of sample geometry and electron beam positioning on magnon spectra in support of the spatially resolved data presented in Fig. 4 of the main manuscript, we performed additional magnon EELS simulations utilising the TACAW method.

Our simulations were conducted on a slab model using a supercell with 11 × 16 × 96 repetitions (of dimensions 4.587 nm × 6.672 nm × 40.032 nm) of the NiO cubic unit cell. We employed the same experimental parameters outlined in the main text for the bulk dispersion curve calculations, including a probe convergence semi-angle of 2.25 mrad. Periodic boundary conditions were applied along the [010] and [001] directions, while a vacuum boundary was introduced along the [100] direction to emulate slab geometry. Given the range of considered exchange interactions (up to 5.9 Å), a width of the slab of 4.587 nm is sufficient to prevent magnetic exchange interactions of the two surfaces as well as to reach a bulk-like behaviour in the central region. A fixed sample thickness of 40 nm was maintained for all configurations to ensure consistency.

Two distinct electron beam positions were analysed: the slab centre (wherein the beam was centred within the slab's xy-plane), and the slab's edge (wherein the beam was positioned inside the slab, 2.1 Å away from the boundary of the slab's xy-plane). The resulting spectra, depicted in Supplementary Figs. 7 and 8 are compared to the bulk NiO spectral features from the main text. Key observations include:

- **a reduced intensity at the slab's edge**. The magnon signal intensity at the edge of the slab was significantly reduced, by a factor of approximately 3-4 compared to the centre of the slab. This reduction can be attributed to the limited spatial overlap of the beam with the slab's magnonic modes as well as to local modifications of the magnon response in this region.
- **an energy redshift at the slab's edge**. Magnons near the slab's edge exhibited a redshift, consistent with localised variations in magnonic interactions influenced by boundary conditions.

These findings underscore the sensitivity of magnon spectra to both sample geometry and beam positioning. We note that due to computational limitations these EELS simulations do not fully reproduce the geometry of the spatially resolved experiments (which would have required averaging over a significantly large number of beam positions, requiring excessive computing times). Nevertheless, the simulations highlight the predicted capability of EELS to probe localised magnonic variations, emphasising its potential for studying nanoscale magnetic heterogeneities in slab systems.

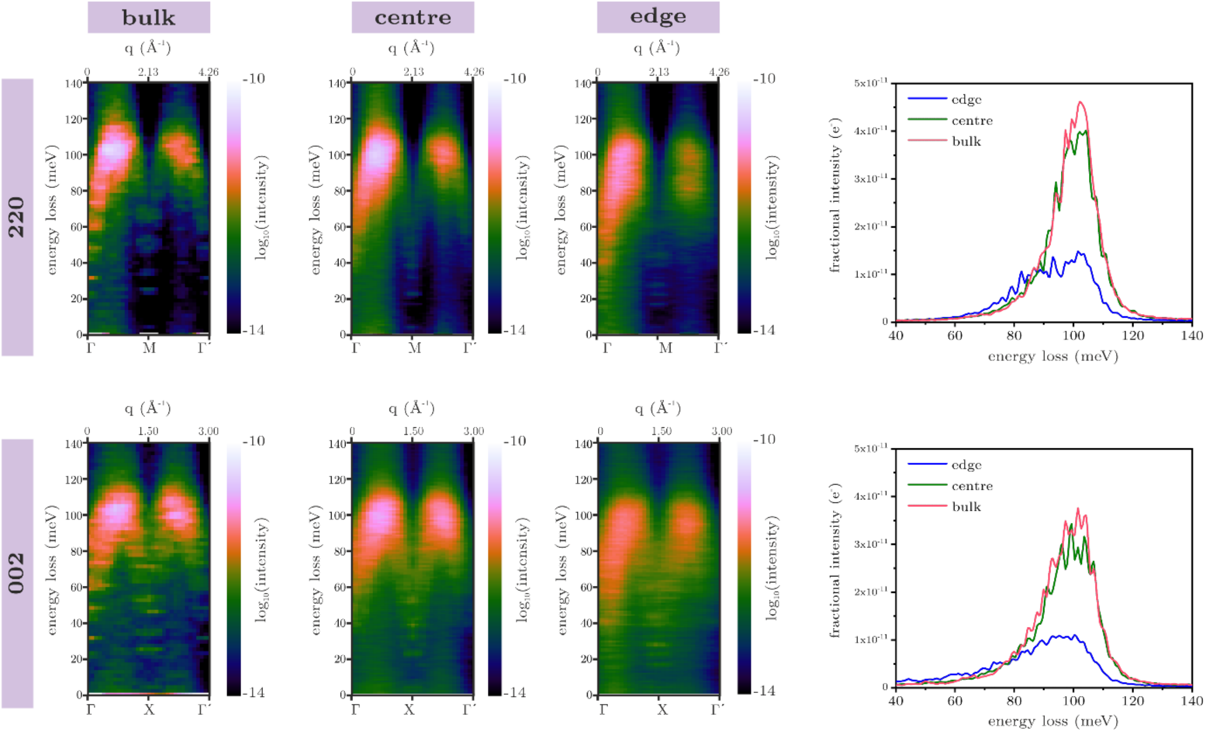

Supplementary Fig. 7 | Spatial dependence of magnon scattering across a NiO slab. *Simulated magnon EELS dispersions along the Γ → M and Γ → X* ***q****-paths of the Brillouin zone for NiO, at different positions across a 4.587 nm-wide NiO slab surrounded by vacuum. The bulk calculations, as reported in the main manuscript, Fig. 3, are reprised here and labelled 'bulk'. Integrated magnon spectra at a wave-vector of* $q = 1.25$ *Å*$^{-1}$ *show the drop of the magnon signal at the slab's edge compared to its centre, consistent with experimental observations.*

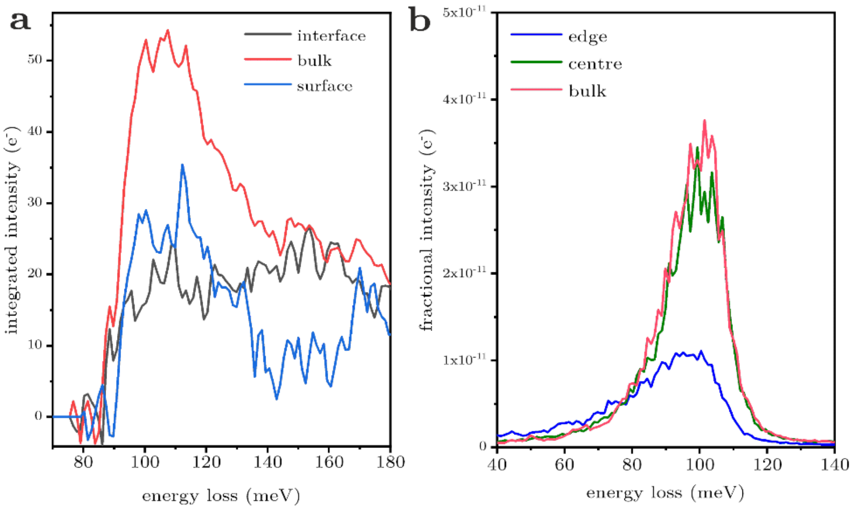

Supplementary Fig. 8 | Comparison between experiment and theory across a NiO thin film. ***a.*** *Background-subtracted magnon spectra from areas of the NiO film marked in Supplementary Fig. 6b.* ***b.*** *Calculated EELS magnon spectra for different positions in the NiO slab (bulk, centre, edge, as indicated).*

Supplementary references

[70] Sun, Q. *et al.* Mutual spin-phonon driving effects and phonon eigenvector renormalization in nickel (II) oxide. *Proceedings of the National Academy of Sciences* **119**, e2120553119 (2022). https://pnas.org/doi/full/10.1073/pnas.2120553119.

[71] Kittel, C. Introduction to Solid State Physics, 8th Edition, Wiley, New York (2005).

[72] Dellby, N., and Batson, P. Private communication.

[73] Fung, K., L., Y. *et al.* Accurate EELS background subtraction – an adaptable method in MATLAB. *Ultramicroscopy* **217**, 113052 (2020). https://doi.org/10.1016/j.ultramic.2020.113052.

[74] Haas, B. *et al.*, Atomic-Resolution Mapping of Localized Phonon Modes at Grain Boundaries. *Nano Lett.* **23**, 5975–5980 (2023). https://doi.org/10.1021/acs.nanolett.3c01089.

[75] Hachtel, J. A., Lupini, A., R., and Idrobo, J. C. Exploring the capabilities of monochromated electron energy loss spectroscopy in the infrared regime. *Scientific Reports* **8**, 5637 (2018). https://doi.org/10.1038/s41598-018-23805-5.

[76] do Nascimento, J., A. *et al.*, Confined magnon dispersions in ferromagnetic and antiferromagnetic thin films in a second quantization approach: the case of Fe and NiO. *Phys. Rev. B.* **110**, 024410 (2024). https://doi.org/10.1103/PhysRevB.110.024410.

[77] Cousland, G. P., Cui, X., Y., Ringer, S., Smith, A., E., Stampfl, A., P., J., and Stampfl, C., M. Electronic and vibrational properties of yttria-stabilised zirconia from first-principles for 10–40 mol% Y_2O_3. *Journal of Physics and Chemistry of Solids* **75**, 1252-1264 (2014). https://doi.org/10.1016/j.jpcs.2014.05.015.

[78] Qi, R. *et al.* Measuring phonon dispersion at an interface. *Nature* **599**, 399-403 (2021). https://doi.org/10.1038/s41586-021-03971-9.
